# Buffalo Hospital of the Sisters of Charity

**Published:** 1851-02

**Authors:** 


*Buffalo Hospital of the Sisters of Charity.*—By the "first general report of the Trustees and managers" of this Institution, we learn that up to the time of making the report, Nov. 27, relief had been extended to 1513 sick. The following financial statement is copied from the Report:—

The Hospital was begun in 1848. In July of that year, a lot and a house sadly wanting repairs, were purchased for $3,700; other lots at an expense, interest, &c., computed, of $3,375, were purchased soon after. The first repairs of the house, fencing, &c., cost $1,300; a first addition with baths, &c., was erected at an expense of $1,700; a second addition, large and commodious, with furnaces, a room for the surgical operations, &c., cisterns, &c., was put up at an expense of $5,400; the dead house and stable cost $200; beds, bedding, furniture, medicines, surgical instruments, provisions, &c., cost $9,192; blinds, repairs, articles recently purchased, amount to $350; servants' hire for two years and three months, cost about $600; making a total of $25,817. To meet this expense a collection was made in Buffalo, many of all religious denominations kindly contributing, it produced $980; donations by visitors amount to about $300; the Fair of 1848, yielded $1,016; receipts from patients, or from those who charitably paid for them, amount to $2,666; the State Legislature generously granted $9,000; for the county poor, $577 has been received; Bishop Timon collected from his friends out of this Diocess, $3,100; a citizen of Buffalo has donated $2,600; making the aggregate sum received, $20,579. There is due to the Hospital by the Commissioners of Emigration, about $340. The Hospital yet owes $3,800 on the lots and house; $1,000 has been borrowed to meet current expenses, and some small debts, not exceeding $500, are due through the city.

The following extract exhibits the revenue derived from patients:—

Since the opening of the Hospital 605 patients have paid for themselves generally at the rate of $1,50 per week; 823 have been charity patients; many were classed among pay patients, who, their funds giving out, really paid but for a part of their stay; thus 79 persons, classed as pay patients, stayed 1603 weeks and 3 days in the Hospital, yet they could only pay for 126 weeks and 1 day, leaving the greater part, viz., 1477 weeks, on charity.

A fair has been held since the publication of the Report, the net proceeds of which were fifteen hundred dollars.

The following are the medical officers of the Institution:

From April 1st to Oct. 1st.

*Attending Physicians*—Dr. Mackay, and Dr. Geo. N. Burwell.

*Consulting Physicians*—Dr. G. F. Pratt, and Dr. I Barnes.

*Attending Surgeon*—Dr. A. S. Sprague.

*Consulting Surgeon*—Dr. J. G. Camp.

From Oct. 1st to April 1st.

*Attending Physician*—Dr. Austin Flint.

*Consulting Physician*—Dr. E. Wallis.

*Attending Surgeon*—Dr. F. H. Hamilton.

*Consulting Surgeon*—Dr. James P. White.

*House Students*—Sandford Eastman, and E. H. Gibbs.

